# Effects of 275 nm Ultraviolet Light-Emitting Diode Irradiation on Oral Bacteria In Vitro and Toothbrush Sanitization

**DOI:** 10.3390/microorganisms14061322

**Published:** 2026-06-12

**Authors:** Qing Liu, Jia Chen Li, Simin Peng, Cynthia Kar Yung Yiu, Hai Ming Wong

**Affiliations:** Paediatric Dentistry and Orthodontics, Faculty of Dentistry, The University of Hong Kong, Hong Kong SAR 999077, China; liuq1029@connect.hku.hk (Q.L.); jiachenl@connect.hku.hk (J.C.L.); pengsm@hku.hk (S.P.); ckyyiu@hku.hk (C.K.Y.Y.)

**Keywords:** ultraviolet irradiation, UVC, oral bacteria, toothbrush sanitization

## Abstract

The oral cavity harbors a complex microbial community where pathogens implicated in dental caries and periodontitis can heavily colonize toothbrushes, transforming them into persistent sources of contamination that threaten both oral and systemic health. Consequently, this study evaluated the bactericidal efficacy of 275 nm ultraviolet light-emitting diode (UV-LED) irradiation against common oral bacteria in vitro and its practical utility for extraoral toothbrush sanitization. Suspensions of *Streptococcus mutans*, *Streptococcus sanguinis*, *Porphyromonas gingivalis*, and *Fusobacterium nucleatum* were irradiated for 3 min, 6 min, and 9 min. Bacterial growth and bactericidal effects were measured using growth curve and colony-forming unit assays, respectively. LIVE/DEAD staining and crystal violet staining were used to evaluate the bacterial viability and multispecies biofilm formation after irradiation. Additionally, the sanitization effects of a 275 nm UVC-based portable device on used toothbrushes were investigated. Direct UVC irradiation at 275 nm exhibited strong bactericidal effects against common oral bacteria in vitro. UVC irradiation also showed great sanitization effects on used toothbrushes. In summary, the vulnerability of common oral bacteria to 275 nm UVC, combined with its sanitizing efficacy on used toothbrushes, establishes a solid basis for extraoral sanitization, offering a reliable strategy to mitigate the risk of oral pathogen transmission from contaminated toothbrushes.

## 1. Introduction

Ranking only behind the gut, the human oral cavity contains a vast and varied microbial ecosystem, including over 700 species of bacteria along with various microeukaryotes, archaea, and viruses [1]. Common oral bacteria, such as *Streptococcus mutans* (*S. mutans*), *Streptococcus sanguinis* (*S. sanguinis*), *Porphyromonas gingivalis* (*P. gingivalis*), and *Fusobacterium nucleatum* (*F. nucleatum*), are implicated in several oral diseases like dental caries and periodontitis. In recent years, there have been ongoing concerns about innovative antimicrobial strategies for controlling oral bacteria.

Toothbrushing is the most important method for removing plaque and maintaining good oral hygiene. However, toothbrushes can also be contaminated by microorganisms from the oral cavity, human skin, the surrounding environment, storage containers, and aerosols from toilet flushing, which can be hazardous to oral health and even systemic health due to the survival of cariogenic and periodontopathic bacteria [2]. Evidence has shown that approximately 1.42 × 10^6^ to 1.19 × 10^7^ colony-forming units (CFUs) per toothbrush accumulate on used toothbrushes [3]. Therefore, effectively sanitizing toothbrushes after brushing has become increasingly important.

Phototherapy is an alternative treatment, mainly for skin conditions like eczema and vitiligo [4,5]. It often uses ultraviolet (UV) light to reduce inflammation and slow down cell proliferation. In clinical dentistry, UV light has been utilized for disinfection [6], tooth bleaching [7], and detection of dental caries [8]. According to the World Health Organization, the wavelength of UV light ranges from 100 to 400 nm and is divided into three bands: UVA (315–400 nm), UVB (280–315 nm), and UVC (100–280 nm). UVA and UVB irradiation can suppress the immune system and reduce inflammatory responses, serving as a therapeutic modality for various inflammatory skin diseases [9]. UVC has potent antibacterial capability, causing cell death by altering the chemical structure of bacterial DNA, as the germicidal action range is around 250–270 nm [10]. UVC irradiation has been reported in anti-oral cancer therapy [11], decontamination of dental implants [12], and sterilization of dental devices [13,14]; however, its use for toothbrush sanitization is still in early development. One systematic review highlighted promising but heterogeneous evidence, with variable irradiation parameters, inconsistent outcome measures, and limited validation [15]. Our aims were therefore to investigate the effect of 275 nm UVC light-emitting diode (UVC-LED) irradiation on the oral bacteria in vitro, as well as its effectiveness in extraoral toothbrush sanitization.

## 2. Materials and Methods

### 2.1. Bacterial Strains and Growth Conditions

To evaluate the efficacy of UVC against key oral disease-related pathogens for potential intraoral application, *S. mutans* (ATCC 700610), *S. sanguinis* (ATCC 10556), *P. gingivalis* (W83), and *F. nucleatum* (CCUG 9126) were used in this study. *S. mutans* and *S. sanguinis* were incubated in brain−heart infusion broth (BHI) under anaerobic conditions (85% N_2_, 10% H_2_, and 5% CO_2_) at 37 °C, while *P. gingivalis* and *F. nucleatum* were incubated in Pg Broth containing 30 g/L trypticase soy broth, 5 g/L yeast extract, 5 mL/L hemin, and 0.5 μg/L vitamin K under the same conditions.

### 2.2. UV-LED Light Source

The small UV-LED (3.5 × 3.5 × 1.6 mm) used in this study was powered by two 1.5 V AA alkaline batteries. According to the manufacturer’s specifications, the forward voltage of the UVC-LED ranged from 5.5 to 7.0 V, and the reverse current was 10 μA. The manufacturer-provided power output was 30 mW, yielding an irradiance (power density) of 42 mW/cm^2^. The exposure amount at each irradiation time was calculated as *E* = *P* × *t*, where *E* is the energy density (dose) in mJ/cm^2^, *P* is the irradiance in mW/cm^2^, and *t* is the time in seconds [16,17]. Based on previous studies investigating the bactericidal effects of UV light on oral bacteria [16,17] and our preliminary pilot results, specific irradiation intervals (3, 6, and 9 min) were selected. Different bacterial suspensions contained within a 96-well plate underwent UVC-LED irradiation for these specific durations from a 2 mm proximity. Non-irradiated bacteria served as the control group. The data are presented in Table 1.

### 2.3. Growth Curve of the Irradiated Bacteria

All bacteria cells were harvested by centrifugation (5000× *g* for 10 min) and washed once with sterile phosphate-buffered saline (PBS). *S. mutans* and *S. sanguinis* were irradiated at a concentration of 1 × 10^6^ CFU/mL for different times (3, 6, 9 min), and then inoculated at a dilution of 1:10 into fresh BHI medium. Bacterial growth was measured every 30 min until 24 h using a microplate reader (CLARIOstar, BMG LABTECH, Ortenberg, Germany) at 600 nm. *P. gingivalis* and *F. nucleatum* were irradiated at a concentration of 1 × 10^7^ CFU/mL, and then inoculated at a dilution of 1:10 into fresh Pg broth. Bacterial growth was measured every 12 h until 48 h at 600 nm. The experiment was repeated three times, and the representative growth curves were plotted.

### 2.4. CFU Assay

All bacteria cells were harvested by centrifugation (5000× *g* for 10 min), washed once with PBS, and then resuspended in BHI or Pg broth at a concentration of 1 × 10^7^ CFU/mL. Bacterial suspensions of 100 μL were added into dark 96-well plates and irradiated by the UV-LED for different times (3, 6, 9 min). After irradiation, the bacterial suspensions were diluted and seeded on the horse blood agar plates, and then incubated under anaerobic conditions for 2–4 days. The bacterial density was determined by counting the colonies and recording the values as CFU/mL. The bactericidal effects were defined as % viability calculated as follows: % viability = 100% × CFU_(3,6,9 min irradiation)_/CFU_(control group)_.

### 2.5. Bacterial Viability Test

To determine the viability of different strains following exposure to 275 nm UVC light, the LIVE/DEAD BacLight Bacterial Viability Kit (Thermo Fisher Scientific, Waltham, MA, USA) was utilized. After irradiation at a concentration of 1 × 10^7^ CFU/mL, each bacterial suspension was transferred into a new dark microtube and stained with SYTO 9/PI staining solution for 30 min according to the manufacturer’s instructions. Fluorescence images were obtained using confocal laser scanning microscopy (CLSM, LSM 900, Zeiss, Oberkochen, Germany). The fluorescence intensity of live and dead bacteria cells was analyzed by ImageJ software (National Institutes of Health, version 1.53, Bethesda, MD, USA).

### 2.6. Crystal Violet Staining of Multispecies Biofilm

The crystal violet staining assay was used to evaluate the effect of UV irradiation on the biofilm formation. The four bacterial strain suspensions (in 1:1:1:1 ratio in Pg broth) were irradiated and then incubated for 3 days at 37 °C under anaerobic conditions to construct the multispecies biofilm. After removing the planktonic bacteria and washing twice with sterile PBS, the multispecies biofilm was stained with 200 μL 0.1% crystal violet solution for 15 min at room temperature. Then, the biofilm was washed three times with sterile PBS to remove the excess crystal violet dye. Bound crystal violet was released by adding 95% ethanol solution into the multispecies biofilm. The absorbance at 575 nm was determined using a microplate reader (SpectraMax iD5, Molecular Devices, San Jose, CA, USA). The experiments were performed in triplicate and repeated three times.

### 2.7. Preliminary Evaluation of Toothbrush Sanitization After UV-LED Irradiation

**Figure 1:**
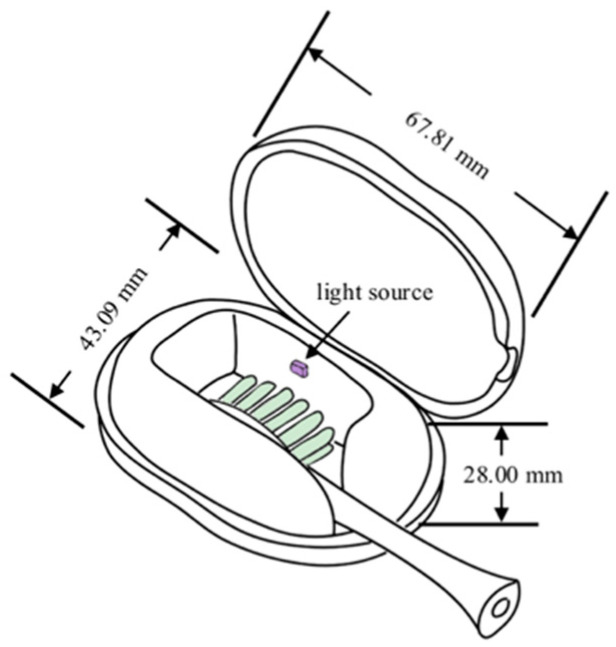
The portable toothbrush sterilization device utilized for extraoral sanitization. The device features an oval-shaped design with dimensions of 67.81 mm (length) × 43.09 mm (width) × 28.00 mm (height). The built-in 257 nm UVC-LED light source is specifically positioned approximately 2 mm away from the toothbrush head.

To explore the potential impact of UV irradiation on toothbrush sanitization, a preliminary evaluation of the bactericidal effects of UV irradiation on the used toothbrushes was conducted utilizing a portable toothbrush sterilization device (OralShield Pro UV Toothbrush Sanitizer, UPC: 8-50066-64630-4, HJS Creations, North Muskegon, MI, USA). The device features an oval-shaped design with a designated space for the toothbrush head. Its dimensions are 67.81 mm in length, 43.09 mm in width, and 28.00 mm in height (Figure 1). Forty-two subjects were randomly selected and all met the inclusion criteria, which was approved by the Institutional Review Board of The University of Hong Kong/Hospital Authority Hong Kong West Cluster (IRB No. UW 24-562). Subjects who had good oral health (i.e., no dental caries or periodontitis), could brush their teeth independently, and who had signed the written consent form, were enrolled in this study. All participants were provided with two identical toothbrushes and toothpastes, and were trained on the Bass method of tooth brushing. After one week, the first set of used toothbrushes were collected and immersed in 20 mL of sterile normal saline solution. After ultrasonication for 5 min at room temperature, the suspensions were diluted and seeded on the horse blood agar plates. Subsequently, the CFUs were counted to establish the baseline for toothbrush bacteria for each individual. Following another week, the second set of used toothbrushes were collected and immersed in 20 mL of sterile normal saline solution after being irradiated with 275 nm UV for 9 min. The CFUs were then counted after the same treatment and culture condition. The effect of UV irradiation on toothbrush sanitization was assessed by % viability, calculated as follows: % viability = 100% × CFU_(irradiation)_/CFU_(baseline)_.

### 2.8. Statistical Analysis

The statistical data analysis was carried out using SPSS software (version 28, IBM Corporation, Armonk, NY, USA). The evaluation of toothbrush sanitization after UV irradiation was statistically analyzed using the *t*-test, while other experiments were analyzed for statistical significance using analysis of variance (ANOVA) and Dunnett’s tests.

## 3. Results

### 3.1. Growth Inhibition of Oral Bacteria After 275 nm UV Light Irradiation

**Figure 2:**
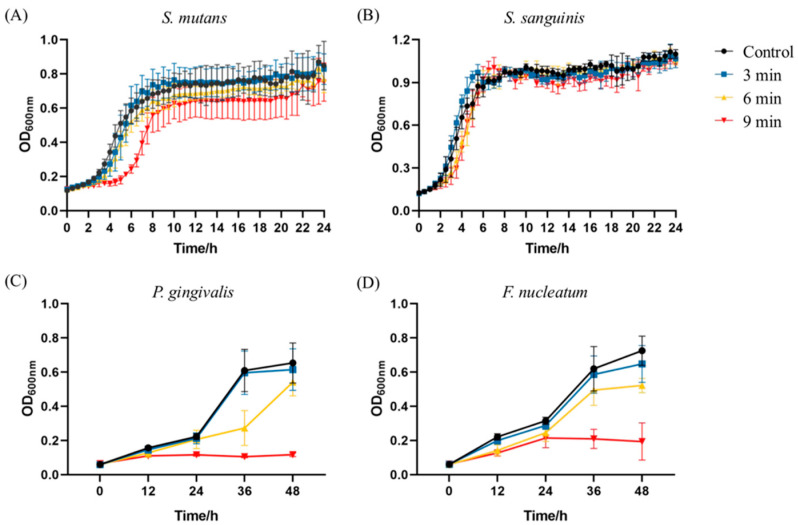
Effect of 275 nm UV irradiation on the growth of oral bacteria. Planktonic suspensions of *S. mutans* (**A**), *S. sanguinis* (**B**), *P. gingivalis* (**C**), and *F. nucleatum* (**D**) were exposed to UVC irradiation for 3, 6, and 9 min, and subsequently inoculated into fresh culture medium at 1:10 dilution. Bacterial growth was monitored by measuring the optical density at 600 nm (OD_600nm_) using a microplate reader.

The effects of UV irradiation for different times on the growth of common oral bacteria including *S. mutans*, *S. sanguinis*, *P. gingivalis*, and *F. nucleatum* are shown in Figure 2. UV irradiation resulted in significant growth inhibition of *S. mutans*, *P. gingivalis*, and *F. nucleatum*. The growth curve assay demonstrated that UV irradiation had a time-dependent inhibitory effect on the growth of planktonic *S. mutans*, with a prolonged logarithmic growth phase and a decreased absorbance value at the plateau compared with the control group (Figure 2A). Following the addition of BHI after 4 h, *S. sanguinis* groups subjected to 6 and 9 min of irradiation exhibited a marginal decrease in optical density compared to the untreated control. However, *S. sanguinis* in all UV-irradiated groups showed a similar level of optical density as the control group after adding BHI for 8 h (Figure 2B). With 6 min and 9 min of irradiation, the growth of *P. gingivalis* and *F. nucleatum* was clearly inhibited. After irradiation for 6 min and 9 min, the optical density of *P. gingivalis* and *F. nucleatum* initially increased temporarily, followed by a growth inhibitory phase, showing significantly lower OD_600_ values compared to the control culture (Figure 2C,D).

### 3.2. Bactericidal Effects of 275 nm UV Light on Oral Bacteria In Vitro

**Figure 3:**
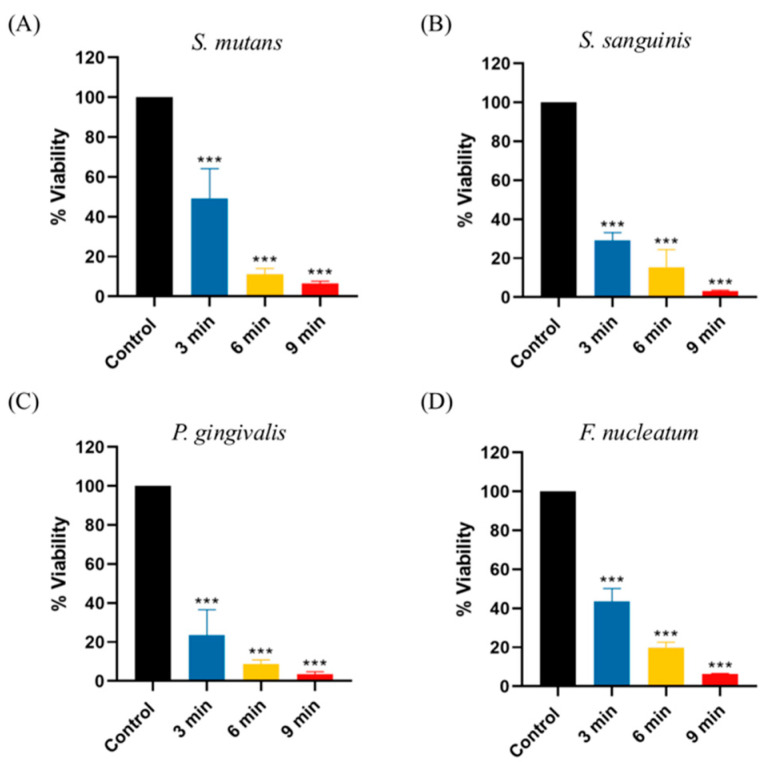
Bactericidal effect of 275 nm UV irradiation against oral bacteria in vitro. Suspensions of *S. mutans* (**A**), *S. sanguinis* (**B**), *P. gingivalis* (**C**), and *F. nucleatum* (**D**) at an initial concentration of 1 × 10^7^ CFU/mL were irradiated for 3, 6, and 9 min. Surviving bacteria were quantified using the CFU assay by seeding the suspensions onto blood agar plates. Colonies on the plates were counted, and the bactericidal effects were expressed as % viability, calculated as follows: % viability = 100% × CFU_(3,6,9 min irradiation)_/CFU_(control group)_. (*** *p* < 0.001 vs. Control).

Significant bactericidal effects from 275 nm UV irradiation were observed for four bacteria compared with the control group (Figure 3). With exposure to 275 nm UV irradiation for 9 min, the viability of all four bacteria was decreased to below 10% (Figure 3). The viability of *S. mutans* was reduced to 49.21% and 11.15% after being irradiated for 3 min and 6 min, respectively (*p* < 0.001, Figure 3A). Compared with the control group, the viability of *S. sanguinis* decreased by 70.81%, 84.63%, and 96.89% after irradiation for 3 min, 6 min, and 9 min, respectively (*p* < 0.001, Figure 3B). Irradiation with 275 nm UV for 3 min, 6 min, and 9 min resulted in viability reductions in *P. gingivalis* to 22.58%, 8.59%, and 3.30%, respectively (*p* < 0.001, Figure 3C). Moreover, when exposed to 275 nm UV irradiation for 3 min, 6 min, and 9 min, 59.27%, 82.02%, and 93.51% of *F. nucleatum* were killed, respectively (*p* < 0.001, Figure 3D).

**Figure 4:**
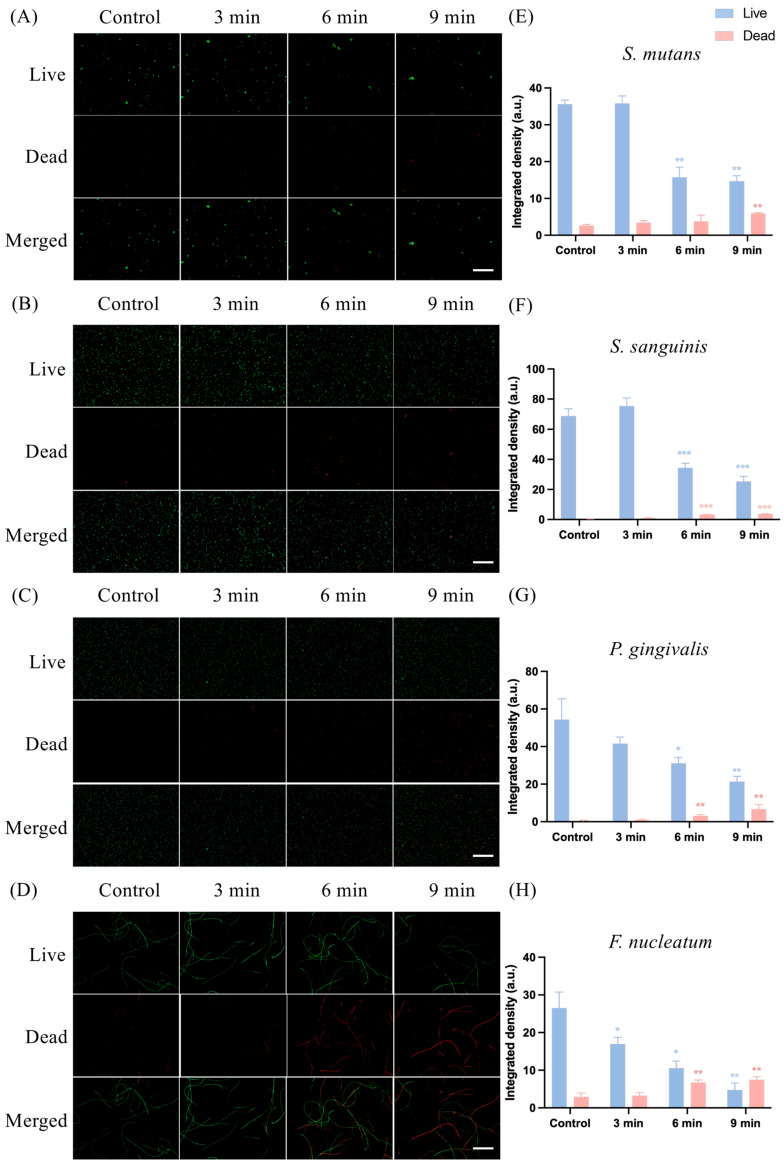
Bacterial viability assessment of oral bacteria following 275 nm UVC irradiation using LIVE/DEAD staining. Representative fluorescence microscopy images of *S. mutans* (**A**), *S. sanguinis* (**B**), *P. gingivalis* (**C**), and *F. nucleatum* (**D**) planktonic cells after 3, 6, and 9 min of UVC exposure. Live bacterial cells were stained green, while dead cells were stained red. Scale bar = 50 µm. (**E**–**H**) Quantification of the integrated fluorescence intensity for each bacterial strain was performed using ImageJ software. The abscissa represents the group name, and the ordinate represents the integrated fluorescence intensity. (*** *p* < 0.001 vs. Control, ** *p* < 0.01 vs. Control, * *p* < 0.05 vs. Control).

The bactericidal effects of UV irradiation on oral bacteria were also observed using LIVE/DEAD cell viability assay with CLSM. Living cells were indicated by green fluorescence, while dead cells were indicated by red fluorescence. The results demonstrated that UV irradiation resulted in bacterial cell death in a time-dependent manner (Figure 4). After UV irradiation for 6 min and 9 min, the number of live bacteria of *S. mutans*, *S. sanguinis*, *P. gingivalis*, and *F. nucleatum* was significantly decreased, while the number of dead bacteria clearly increased (Figure 4A–D). Similarly, quantification results from LIVE/DEAD assay demonstrated that with irradiation time, the integrated green fluorescence intensity gradually decreased, while the integrated red fluorescence intensity gradually increased (Figure 4E–H). These findings indicate the bactericidal effects of UV irradiation on oral bacteria.

### 3.3. Prevention of Biofilm Development In Vitro

**Figure 5:**
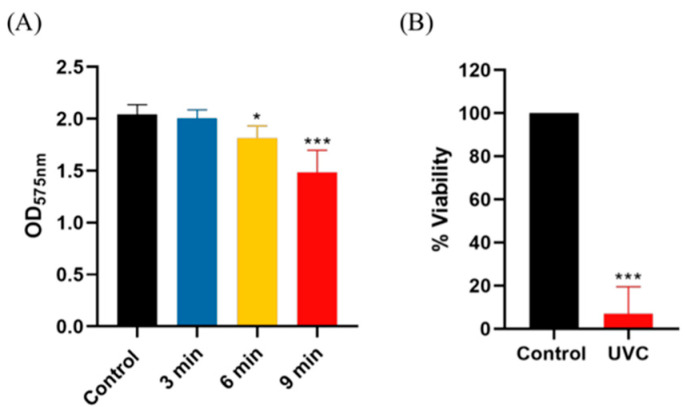
The preventive effect of 275 nm UV irradiation on multispecies biofilm formation and its application in toothbrush sanitization. (**A**) Prevention of multispecies biofilm development. A mixed suspension of the four oral bacterial strains (1:1:1:1 ratio) was pre-irradiated for 3, 6, and 9 min prior to anaerobic incubation. Total biofilm biomass was quantified using the crystal violet staining assay by measuring the absorbance at 575 nm (OD_575nm_). (**B**) Extraoral sanitization efficacy on used toothbrushes. Used toothbrushes collected from participants were ultrasonicated to detach microorganisms. The bacterial load was quantified via CFU assay before (baseline) and after 9 min of UV irradiation using the portable device. The sanitization effect was expressed as % viability = 100% × CFU_(irradiation)_/CFU_(baseline)_. (*** *p* < 0.001 vs. Control, * *p* < 0.05 vs. Control).

Crystal violet staining revealed the influence of UV irradiation on multispecies biofilm formation. As shown in Figure 5A, biofilm formation in the 3 min exposure group remained comparable to the control (*p* > 0.05). Irradiation for 6 min and 9 min significantly prevented the formation of multispecies biofilm compared with the control group (*p* < 0.05 and *p* < 0.001, respectively). The results suggested that 275 nm UV irradiation was also effective for prevention of multispecies biofilm development.

### 3.4. The Sanitization Effect of UV Irradiation on Used Toothbrushes

Based on the above bactericidal results, the efficacy of toothbrush sanitization by irradiation for 9 min utilizing the portable toothbrush sterilization device was evaluated. Used toothbrushes were collected from subjects, and CFU assay was performed after UV irradiation. The preliminary results showed that UV irradiation had a bactericidal effect on toothbrush-derived bacteria (Figure 5B). After 9 min of UV irradiation, the toothbrush-derived bacteria exhibited an average reduction of 2.03 log_10_ CFU/mL, corresponding to a decrease in average viability to 6.90% (*p* < 0.001, Figure 5B). The finding indicated the device had great sanitization effects on the used toothbrushes.

## 4. Discussion

In the present study, we found that a 9 min exposure to 275 nm UVC light exhibits potent antimicrobial efficacy against common oral bacteria responsible for dental caries and periodontitis in vitro. In addition, a portable toothbrush sterilization device utilizing this wavelength showed great efficiency in sanitizing toothbrushes.

Numerous studies have demonstrated the bactericidal effects of UV light. Although UV light has been increasingly applied in dental tool disinfection, tooth whitening, dental caries detection, and dental implant pretreatment [12,18,19], its use in toothbrush sanitization is limited due to variable irradiation parameters, inconsistent outcome measures, and limited validation. In our study, the forward voltage of the UVC-LED was 5.5–7.0 V, and the reverse current was 10 μA. The power of the UVC-LED was 30 mW, and the power density (irradiance) was 42 mW/cm^2^. The energy densities at 275 nm using the UVC-LED for 3, 6, and 9 min were 7560, 15,120, and 22,680 mJ/cm^2^, respectively. Previous studies have shown that various wavelengths of UV light have bactericidal effects on oral bacteria. Irradiation with the 310 nm UVB-LED at 105 mJ/cm^2^ for 1 min showed 30–50% bactericidal activity against *S. mutans*, *S. sanguinis*, *P. gingivalis*, and *F. nucleatum* [16]. Another in vitro study found that the viability of oral bacteria significantly decreased with 285 nm UV irradiation, and the viability of *Enterococcus faecalis* was significantly reduced after 5 min of irradiation in a curved simulated root canal [20]. Direct 265 nm UVC-LED irradiation at 3.2 J/cm^2^ for 5 min showed strong bactericidal effects on *S. mutans* in vitro [17]. Therefore, the irradiation time and wavelength are considered to play crucial roles in the antibacterial effects. Based on these studies and our own preliminary pilot results, 3, 6, and 9 min were chosen as different time points to explore the bactericidal effects of the 275 nm UVC-LED.

In this study, 275 nm UVC-LED irradiation exhibited significant growth inhibition of *S. mutans*, *P. gingivalis*, and *F. nucleatum*, but not *S. sanguinis*, in a time-dependent manner. The slight reduction in the optical density of *S. sanguinis* after irradiation was followed by comparable levels to the control group, which may be attributed to the complex molecular mechanisms of *S. sanguinis*. These mechanisms likely enhance its resilience, making it a strong competitor against other bacteria and a representative in the oral health-related microbiome [21]. Furthermore, UVC irradiation also demonstrated over 90% bactericidal efficacy against *S. mutans*, *S. sanguinis*, *P. gingivalis*, and *F. nucleatum* with a direct 9 min exposure. The results reported in the current study are similar to previously reported results [17]. Additionally, it was revealed by fluorescence microscopy analysis that oral bacterial cell death was induced by UVC irradiation in a time-dependent manner. These findings suggest that 275 nm UVC had both antimicrobial and growth inhibitory effects. As a cost-efficient antimicrobial approach, UVC irradiation can induce DNA damage by generating cyclobutane–pyrimidine dimers and 6–4 photoproducts, resulting in changes in bioactive cell properties [22]. In addition to DNA damage, ROS production and protein alteration also contribute to bacterial cell death [9]. The demonstrated strong bactericidal effects of 275 nm UVC against common oral bacteria highlight its potential in intraoral infection control and extraoral sanitization; however, comprehensive future investigations, particularly regarding mucosal safety, are strictly necessary before any intraoral application can be considered.

While our initial monospecies assays provided the baseline validation for the bactericidal activity of 275 nm UVC, oral bacteria naturally exist in complex communities that are typically more resistant to antimicrobial treatments. To address this, we preliminarily evaluated UVC efficacy against a four-species in vitro biofilm, demonstrating that 6 and 9 min of irradiation significantly prevented multispecies biofilm development. Building upon these findings, we advanced to investigate the extraoral sanitization effects of 275 nm UVC irradiation on used toothbrushes, as several studies have revealed that bacterial contamination with various microorganisms exists on used toothbrushes [3]. Notably, in addition to aerobic bacteria, anaerobic bacteria related to different oral diseases were detected on used toothbrushes, such as *P. gingivalis* and *F. nucleatum* [23]. As a reservoir for microorganisms, contaminated toothbrushes play a crucial role in facilitating disease transmission and increasing the risk of infection [24]. It is necessary to employ effective sanitization methods to reduce toothbrush contamination after use. Evidence indicated that UV light was one of the most efficient domestic options for sanitizing contaminated toothbrushes [25]. Therefore, based on the antibacterial effects of 275 nm UVC irradiation in vitro, we examined the viability of toothbrush-derived bacteria before and after using a 275 nm UVC-based toothbrush sanitization device. Our results showed that the average viability of bacteria derived from used toothbrushes decreased to 6.90% after 9 min of irradiation, which is higher than the reductions observed in other studies [25]. Although our in vitro antibacterial, multispecies biofilm, and toothbrush sanitization assays provide a foundation for evaluating UVC efficacy against complex microbial communities, further comprehensive studies are warranted to more precisely elucidate the impact of UVC on these highly intricate biological systems [26,27,28].

Despite promising sanitization efficacy, several practical considerations regarding the use of UVC light must be addressed. First, direct UVC exposure poses significant risks to human skin and eyes, necessitating safety measures such as shielding and light-blocking features to prevent any accidental user exposure during operation [29]. Second, although the 275 nm wavelength is generally outside the primary ozone-generating spectrum (typically below 240 nm) [30], the potential for trace ozone generation and its subsequent impact on respiratory health warrant further investigation. Finally, repeated UVC irradiation may lead to the photochemical degradation of toothbrush bristle polymers, potentially causing discoloration or increased brittleness over time [31]. Therefore, future evaluations of UVC sanitizers should consider these safety and material integrity factors alongside antimicrobial efficacy.

There are several limitations to this study. First, our investigation focused only on four common oral bacterial strains, without including a broader range of species. Second, because many aerobic bacteria remain on used toothbrushes when exposed to air, which were not included in this experiment, the overall sanitization efficacy of UVC-LED irradiation may not be precisely evaluated. Additionally, we also did not test the eradication of preformed biofilms, a condition that is highly relevant to used toothbrush bristles. Third, this study lacked a direct comparison between the portable UVC device and conventional toothbrush disinfection methods (e.g., chemical disinfectants or home remedies), which limits the assessment of its relative advantages. Fourth, the lack of real-time temperature monitoring during irradiation prevents the complete exclusion of minor photothermal influences. Future studies should incorporate precise thermal monitoring to comprehensively evaluate and isolate the photochemical efficacy of 275 nm UVC in both intraoral and extraoral applications. Finally, given the in vitro nature of this study, thorough follow-up clinical trials are required to comprehensively assess the effectiveness of the UVC device for toothbrush sanitization. Additionally, while UVC generally poses a low risk of inducing rapid genetic resistance, the long-term effects of sub-lethal exposure on bacterial DNA repair mechanisms and potential adaptive tolerance remain to be elucidated. Moreover, rigorous evaluation of in vivo safety and efficacy is warranted before any direct intraoral application can be considered.

## 5. Conclusions

In summary, this study demonstrated the strong bactericidal effects of 275 nm UVC against common oral bacteria and its practical utility in sanitizing used toothbrushes. While limited by the in vitro experimental design and the specific bacterial strains evaluated, these findings provide a basis for its application in routine extraoral sanitization to mitigate the risk of oral pathogen transmission. Furthermore, although this technology holds potential for intraoral infection control, rigorous in vivo safety and efficacy evaluations remain essential before direct clinical applications can be realized.

## Figures and Tables

**Table 1:** Energy density of 275 nm by irradiation with UVC-LED. Power fluencies of UVC-LED were calculated as energy density (mJ/cm^2^) = power density (mW/cm^2^) × time (seconds).

Irradiation Dose (mJ/cm^2^)	3 min	6 min	9 min
275 nm	7560	15,120	22,680

## Data Availability

The original contributions presented in this study are included in the article. Further inquiries can be directed to the corresponding author.

## Abbreviations

The following abbreviations are used in this manuscript:

*S. mutans*	*Streptococcus mutans*
*S. sanguinis*	*Streptococcus sanguinis*
*P. gingivalis*	*Porphyromonas gingivalis*
*F. nucleatum*	*Fusobacterium nucleatum*
CFU	Colony-forming unit
UV	Ultraviolet
LED	Light-emitting diode
PBS	Phosphate-buffered saline
CLSM	Confocal laser scanning microscopy
